# Towards Understanding the Basis of Brucella Antigen–Antibody Specificity

**DOI:** 10.3390/molecules30142906

**Published:** 2025-07-09

**Authors:** Amika Sood, David R. Bundle, Robert J. Woods

**Affiliations:** 1 Complex Carbohydrate Research Center, University of Georgia, Athens, GA 30602, USA; amika@uga.edu; 2 Department of Chemistry, University of Alberta, Edmonton, AB T6G 2R3, Canada

**Keywords:** *Brucella* A and M antigens, *Brucella* monoclonal antibodies, homology modeling, Vina-Carb docking, MD simulations, MM-GBSA binding energy, bound complexes, GLYCAM, AMBER

## Abstract

Brucellosis continues to be a significant global zoonotic infection, with diagnosis largely relying on the detection of antibodies against the Brucella O-polysaccharide (O-PS) A and M antigens. In this study, computational methods, including homology modeling, molecular docking, and molecular dynamics simulations, were applied to investigate the interaction of the four murine monoclonal antibodies (mAbs) YsT9.1, YsT9.2, Bm10, and Bm28 with hexasaccharide fragments of the A and M epitopes. Through stringent stability criteria, based on interaction energies and mobility of the antigens, high-affinity binding of A antigen with YsT9.1 antibody and M antigen with Bm10 antibody was predicted. In both the complexes hydrophobic interactions dominate the antigen–antibody binding. These findings align well with experimental epitope mapping, indicating YsT9.1’s preference for internal sequences of the A epitope and Bm10’s preference for internal elements of the M epitope. Interestingly, no stable complexes were identified for YsT9.2 or Bm28 interacting with A or M antigen. This study provides valuable insights into the mechanism of molecular recognition of Brucella O-antigens that can be applied for the development of improved diagnostics, synthetic glycomimetics, and improved vaccine strategies.

## 1. Introduction

Brucellosis is regarded by the World Health Organization as one of the most serious zoonotic bacterial diseases [1]. It is a costly, highly contagious disease that affects cattle, sheep, goats, pigs, camels, and other productive animals worldwide [2]. It is endemic in a number of countries and costs of the disease worldwide amount annually to several billion dollars. The detection of antibodies against *Brucella* antigens has been at the forefront of the understanding, control, and eradication of brucellosis for over 120 years. Presumptive diagnosis of brucellosis primarily depends on detection, in animal or human sera, of antibodies to the O-polysaccharide (O-PS) component of *Brucella* lipopolysaccharide [2].

Wilson and Miles established in 1932 that the major serological antigens of pathogenic Brucella species were two surface antigens A and M simultaneously expressed on all smooth strains of the bacterium albeit in varying quantitative amounts within a single molecule [3]. Strains designated A^+^M^−^ possessed a preponderance of A over M antigen and M^+^A^−^ strains higher M over A. It is the detection of antibodies to these A and M antigens that forms the basis for the diagnosis of brucellosis [2,4].

In 1939, the A and M antigens were found to contain a polyhydroxyamino compound and formate [5,6]. This insight was confirmed when 4-formamido-4,6-dideoxy-⍺-D-mannopyrannose (Rha4NFo) was isolated as the sole monosaccharide component of the repeating portion of O-PS from *B. abortus* lipopolysaccharide [7]. *Brucella* A dominant strains were originally suggested to be composed of exclusively α1,2-linked D-Rha4NFo residues [7]. *Brucella* M dominant strains were proposed to contain one α1,3-linked D-Rha4NFo for every four α1,2-linked D-Rha4NFo residues [8]. However, it was soon recognized that this model was not complete, and M epitopes were always present in all smooth strains of the pathogen [9].

**Figure 1:**
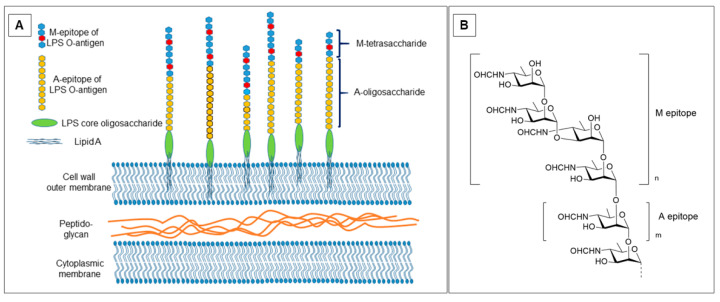
(**A**) Schematic representation of LPS molecules anchored by their Lipid A component in the outer membrane of the Gram-negative coccobacilli, Brucella. The majority of Brucella strains expressing O-polysaccharide have an internal sequence of 1,2 linked 4-formamido-4-deoxy D mannopyranose residues of variable length typically 12–16 residues capped at their reducing end by 1 or 2 copies of M tetrasaccharide. *B. melitensis* 16M may contain as many as 34 4-formamido-4-deoxy-D-mannopyranose residues and include more than 2 copies of capping tetrasaccharide. O-polysaccharides are attached through the core oligosaccharide to Lipid A. (**B**) Definitive structural studies established the structure of the *Brucella* O-antigen with a capping M epitope terminating an A type α1,2-linked D-Rha4NFo polymer, where usually m ≤ 16 [10]. The capping tetrasaccharide occurs a minimum of once, *n* = 1 or may be repeated several times, *n* > 1. Epitope mapping suggests the A epitope likely comprises four or more α1,2-linked D-Rha4NFo residues, e.g., [→2)αRha4NFo(1-]_4_ [13,14]. The M epitope is minimally the disaccharide Rha4NFo(1→3)Rha4NFo but could be as large as the capping tetrasaccharide, [Rha4NFo(1→2)Rha4NFo(1→3)Rha4NFo(1→2) Rha4NFo(1-] [13,14].

Recently the fine structure of *Brucella* O-PS has been re-examined and a revised structure proposed [10] (Figure 1). This work confirmed the original single molecule paradigm of Wilson and Miles. All *Brucella* strains with a smooth LPS (except *B. suis* biovar 2 [11]) essentially consist of two polymeric elements, a 1,2-linked homopolymer that is capped by at least one tetrasaccharide repeating unit containing a α1,3-linked D-Rha4NFo (Figure 1). This latter tetrasaccharide defines the M antigen, while the exclusively α1,2-linked polysaccharide component defines the A antigen. An epitope referred to as C/Y requires from 2 to 4 α1,2-linked D-Rha4NFo residues [12]. It can be appreciated from the general *Brucella* O-antigen structure (Figure 1) that the A antigen is universally present in all smooth *Brucella* with variable expression of the M antigen (Figure 1, m ≥ 2) [10]. The simultaneous expression of two antigenic determinants in a single homopolysaccharide and the recognition of these by antibodies has considerable practical implications in recognition of the antigens by antibodies.

During studies that helped establish the structural basis of the A and M antigens, several monoclonal antibodies, YsT9.1, YsT9.2 Bm10, and Bm28 were developed that proved crucial in linking antibody binding profiles to the fine structures of the two antigens [7,8,9,13,15]. Subsequently the binding sites of these have been subjected to extensive epitope mapping by a large panel of purified polysaccharide antigens and synthetic oligosaccharides [14,15,16,17]. To gain insight into the potential binding modes of these antigens with these distinct monoclonal antibodies, the antibodies have been sequenced and attempts were made to obtain crystal structures of their antigen bound forms. Only one of the four (YsT9.1), an IgG2b [13] with binding profile A >> M yielded a crystal structure without a bound antigen [18] and a rudimentary binding mode was proposed for the interaction with the A antigen [19]. The remaining three antibodies all belonged to the IgG3 subclass, which in our hands failed to provide Fab fragments that crystallized with or without an antigen [Bundle unpublished results]. Two of the three, (Bm10 and Bm28), had binding profiles M >> A [15] and one (YsT9.2) [13] bound A and M antigens with equal affinity [15]. Previously, we have employed a range of experimental approaches, including point mutagenesis [20], chemical modification of the ligands [21], saturation transfer difference NMR spectroscopy [20], x-ray crystallography [21], and deuterium exchange mass spectrometry [22] to characterize antibody paratopes and ligand epitopes, many of which leverage the power of MD simulations to guide experimental design and data interpretation. However homogeneous samples of the A and M epitopes of Brucella O-antigen (Figure 1) are currently unavailable for such experimental work.

This study computationally explores the binding of four murine monoclonal antibodies (mAbs), namely YsT9.1, YsT9.2, Bm10, and Bm28, to hexasaccharide fragments of the A and M epitopes of Brucella O-antigen. By integrating homology modeling, Vina-Carb molecular docking, and molecular dynamics simulations, we attempted to clarify the molecular basis of their differential binding specificities. The docked poses can be further interrogated using extensive experimental epitope mapping data published for these antibodies [14,16,17]. This computational modeling approach aims to overcome a number of unique hurdles to the A and M antigens, including frame shifting between overlapping epitopes and the occurrence of two energetically accessible rotamers of the formamido group [23].

## 2. Results

### 2.1. Docking Analysis

**Figure 2:**
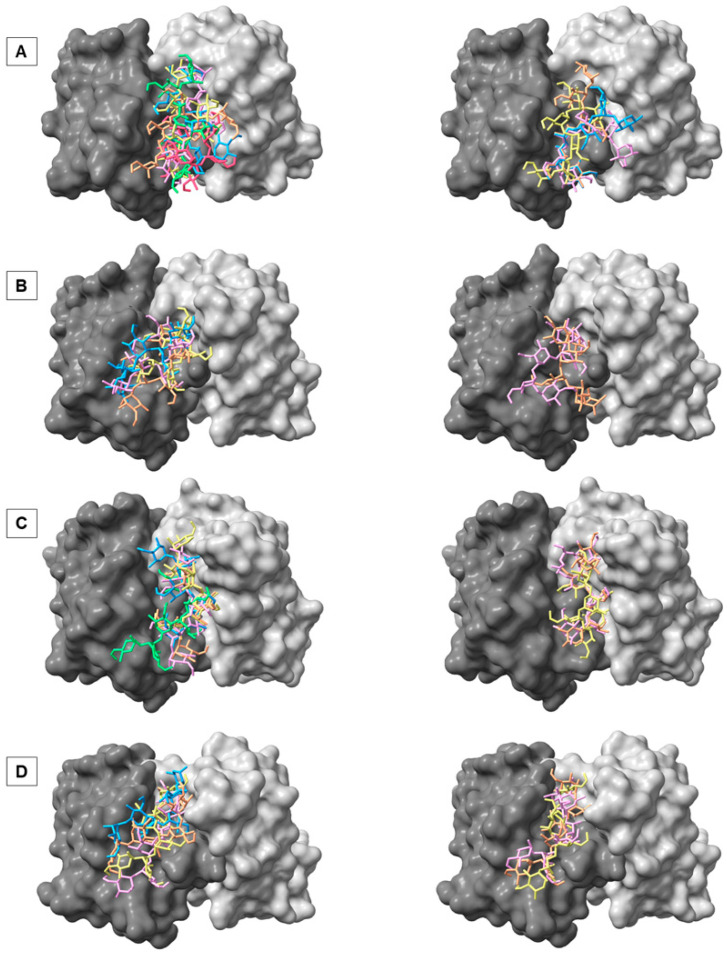
The top docked poses from each cluster (orange, pink, yellow, blue, green, and red) obtained after docking for the A antigen (**left**) and M antigen (**right**) to murine *Brucella* antibodies YsT9.1 (**A**), YsT9.2 (**B**), Bm10 (**C**), and Bm28 (**D**). The antigens are shown as sticks and the antibodies as solvent-accessible surface. The heavy chain of the antibodies is colored dark gray, while the light chain of the antibodies is colored light gray.

To identify the preferred binding sites of the antibody, AutoDock Vina-Carb was used to perform docking of fragments of the A and M epitopes of the O-antigens to the murine antibodies YsT9.1, YsT9.2, Bm10, and Bm28. The docked poses were then divided into clusters using a partitional k-means clustering algorithm. Each docking simulation resulted in a different number of clusters (Table S1) each with a different number of poses. The top ranked pose from each cluster was selected for MD simulation (Figure 2).

### 2.2. Ligand Stability

**Figure 3:**
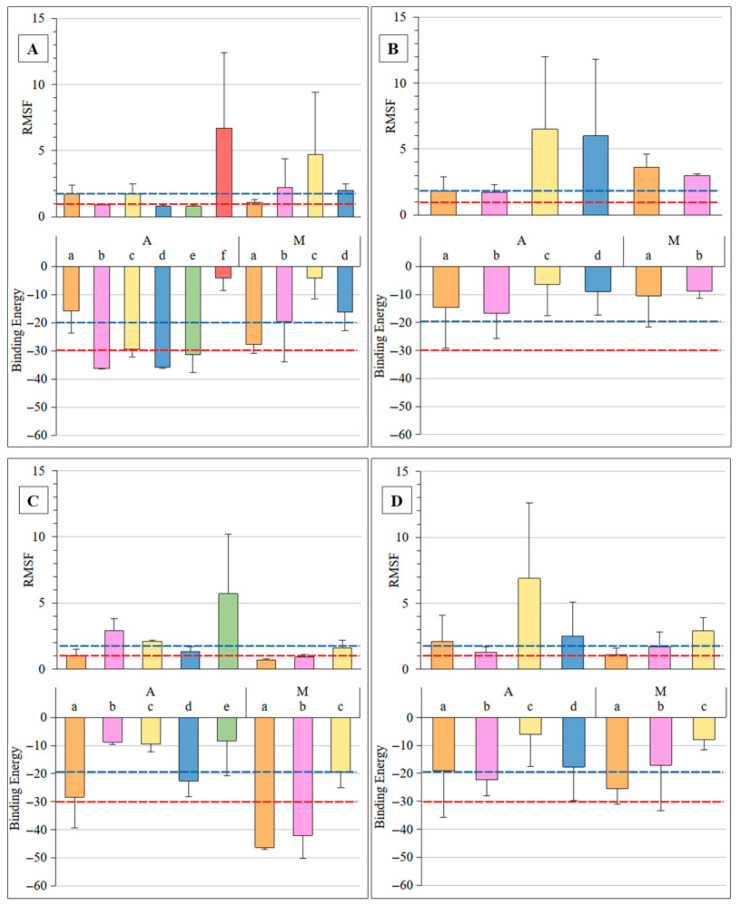
The RMSF (Å) and Binding Energy (kcal/mol) values computed using the last 25 ns of the MD simulations for YsT9.1 (**A**), YsT9.2 (**B**), Bm10 (**C**), and Bm28 (**D**). The A and M antigens are labeled on the *x*-axis and the different clusters (shown structurally in Figure 3) are also indicated alphabetically labeled here (a: orange, b: pink, c: yellow, d: blue, e: green, f: red). The blue lines indicate the median values, and the red lines indicate the cut-off values.

To assess the stability of the docked antigen poses when bound to the antibodies, each complex was subjected to independent molecular dynamics (MD) simulations (100 ns) in triplicate. The Root Mean Square Fluctuation (RMSF) of the antigen atoms was calculated from the final 25 ns of simulation to quantify their mobility within the binding site, with lower RMSF values indicating more stable complexes. The average RMSF values across the three replicates for each antibody–antigen cluster are presented in the top graphs in each panel of Figure 3. Significant variation in antigen mobility was observed across the different antibody–antigen complexes and even among the different docking clusters for the same antibody and antigen.

For example, in panel A representing YsT9.1, most A antigen clusters had relatively low RMSF values (<2 Å), while cluster ‘f’ has an average RMSF of 6.7 Å, breaking away from this trend. Overall, the M antigen clusters exhibited higher fluctuations compared to the A antigen interacting with YsT9.1. Similarly, panel B representing YsT9.2, panel C representing Bm10, and panel D representing Bm28 from Figure 3 showed varied RMSF values across their numerous clusters. The overall RMSF values across all calculated ligand RMSF values ranged from a minimum of 0.6 Å to a maximum of 13.3 Å, with a median of 1.8 Å.

### 2.3. Interaction Energies

The interaction energy for each complex was quantified by calculating the MM-GBSA binding energy with IGB2 parameters, as advised previously [24], from snapshots (2500) taken from the last 25 ns of the MD simulations. Lower (more negative) binding energy values indicate a stronger, more favorable interaction. The average binding energies from the triplicate MD simulation runs for each cluster are presented in the bottom graphs of each panel of Figure 3. Similarly to the RMSF data, the binding energy values displayed considerable variation from complex to complex and across different clusters. For instance, in the bottom graph of panel A of Figure 3 (YsT9.1), several A antigen clusters show favorable (negative) binding energies, while M antigen clusters have less favorable values, consistent with YsT9.1’s preference for the A antigen. In the bottom graph of panel C of Figure 3 (Bm10), some M antigen clusters exhibit highly favorable binding energies compared to A antigens. Across all calculated binding energy values, the range extended from a minimum (most favorable) of −50.8 kcal/mol to a maximum (least favorable) of 1.3 kcal/mol, with a median of −19.2 kcal/mol.

**Figure 4:**
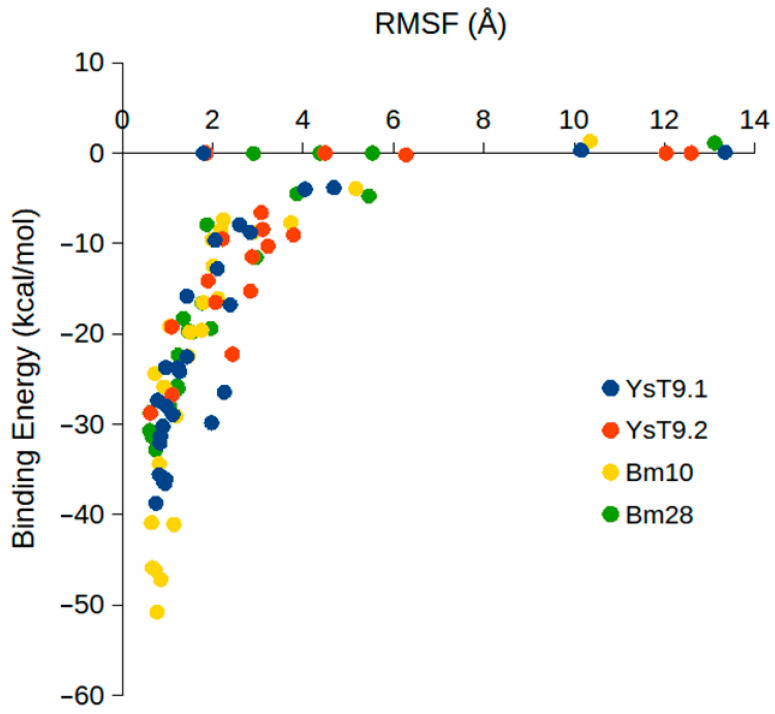
RMSF (Å) versus binding energy (kcal/mol) for all mAb-ligand poses (YsT9.1, YsT9.2, Bm10, and Bm28 shown as blue, red, yellow, and green circles, respectively) simulated in triplicate by MD indicating the strong correlation between low RMSF and strong binding. Complexes with either RMSF below 1 Å or binding energy below −30 kcal/mol were considered stable.

Given that both lower RMSF (indicating higher stability) and lower binding energy (indicating stronger affinity) are indicative of a more favorable interaction, a direct correlation between these two parameters was anticipated. As illustrated in Figure 4, a positive correlation was observed between RMSF and binding energy within a specific range, where increasing stability (decreasing RMSF from approximately 2 Å down to 1 Å) corresponded to stronger binding (decreasing binding energy from approximately 0 kcal/mol down to −30 kcal/mol). Beyond these values, the relationship became asymptotic: very low RMSF values (below ~1 Å) were associated with strongly favorable binding energies (below approximately −30 kcal/mol), while high RMSF values (above ~2 Å) consistently corresponded to binding energies close to 0 kcal/mol, indicative of unstable or non-binding poses. Based on this observed correlation, and to focus on stable interactions, only complexes with ligand RMSF values below 1 Å and binding affinities below −30 kcal/mol in each of the three independent MD runs were considered for further analysis. Clearly, the interaction energies will depend on the calculation method and on the system; however, the RMSF–energy correlation is a useful general feature for developing acceptance criteria. Applying these criteria, the only stable complexes identified were the A antigen bound to YsT9.1 through clusters ‘b’ and ‘d’, and the M antigen bound to Bm10 through cluster ‘a’. These stable interactions are visually evident in Figure 3 as specific bars within Panels A and C falling below both the red dotted line in the RMSF plot (~1 Å) and the red dotted line in the Binding Energy plot (~−30 kcal/mol).

### 2.4. Binding Modes

**Figure 5:**
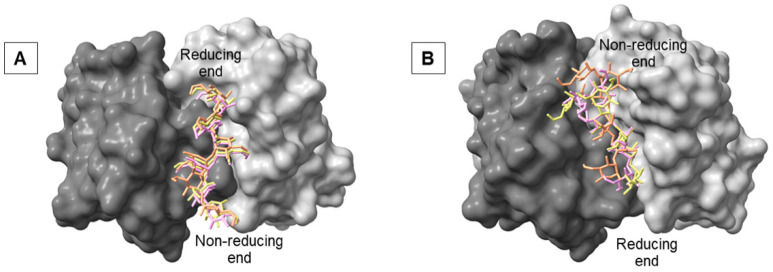
Representative stable-binding modes (orange, pink, yellow) of mAb Ys9.1 in complex with A antigen (**A**) and mAb Bm10 in complex with M antigen (**B**). The antigens are shown as sticks and the antibodies as solvent-accessible surface. The heavy chain of the antibodies is colored dark gray, and the light chain of the antibodies is colored light gray.

To gain a better understanding of how the antigens interact within the identified stable complexes, we analyzed the MD trajectories to identify distinct binding modes. For the stable YsT9.1 antibody complex with A antigen (derived from initial docking clusters ‘b’ and ‘d’), all frames (2500 frames from the last 25 ns) from the three independent MD simulations for each of the two clusters (total six simulations) were merged. Similarly, for the stable Bm10 antibody complex with M antigen (derived from initial docking cluster ‘a’), all frames (2500 frames from the last 25 ns) from its three independent MD simulations were merged. These merged trajectories were then subjected to clustering based on the 3D coordinates of the antigen atoms. This analysis yielded three representative structures for both the YsT9.1-A complex and the Bm10-M complex, representing the most populated conformational states of the antigen within the binding site (Figure 5).

The structural variations among these representative binding modes were assessed by calculating the Root Mean Square Deviation (RMSD) of the ring atoms (C1, C2, C3, C4, C5, O5) of the hexasaccharide antigens. For the YsT9.1-A complex, the three representative structures showed RMSDs ranging from 0.8 Å to 2.1 Å relative to each other (Table S2). As visualized in Figure 5A, these representative structures occupied largely overlapping 3D space within the YsT9.1 binding site. Therefore, these variations were considered to represent minor conformational adjustments around a singular, dominant binding mode. Consequently, for subsequent analyses of the YsT9.1-A interaction, the binding energy values were averaged across all six independent MD simulation runs (three for cluster ‘b’ and three for cluster ‘d’).

For the Bm10-M complex, the three representative structures exhibited larger RMSDs, ranging from 1.4 Å to 3.9 Å (Table S3). Visual inspection (Figure 5B) revealed that two of these representative structures (depicted in yellow and pink) occupied a similar region of the binding site, defining what was termed Binding Mode 1. The third representative structure (depicted in orange) occupied a distinct spatial location, which was designated as Binding Mode 2. Accordingly, for the Bm10-M interaction, binding energy values for Binding Mode 1 were averaged over the two MD simulations whose trajectories were closest to the centroids of the yellow and pink representative structures. The binding energy for Binding Mode 2 was derived from the single MD simulation whose trajectory was closest to the centroid of the third representative structure. This approach allowed us to analyze the energetic contributions separately for these distinct binding orientations observed in the stable Bm10-M complex.

### 2.5. Contribution to Binding from the Perspective of the Antibody

**Figure 6:**
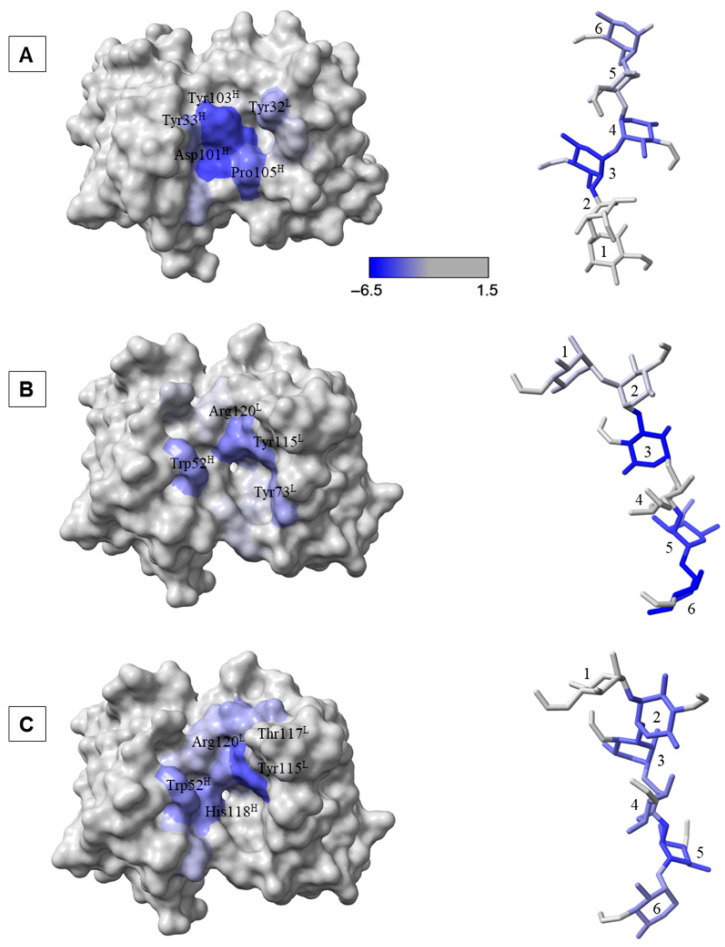
Per-residue binding energy contributions (blue color scale) for the antibody and antigen in the YsT9.1-A complex (**A**), Bm10-M complex in binding mode 1 (**B**), and Bm10-M complex in binding mode 2 (**C**). The antibodies are shown with a solvent-accessible surface (**left**) and the antigen as sticks (**right**). The H and L superscripts indicate heavy chain and light chains, respectively. Antigen residues are numbered 1 through 6, corresponding from the non-reducing to the reducing end.

To identify the amino acid residues of the antibody that are critical for interaction with the antigens (the paratope), the per-residue contributions to the total MM-GBSA binding energy were analyzed. Residues exhibiting favorable contributions stronger than -1.0 kcal/mol were considered. For the YsT9.1 antibody interacting with the A antigen, key residues contributing significantly to the binding were primarily located in the heavy chain, including Tyr32H, Tyr33H, Asp101H, Tyr103H, Pro105H, and Ala106H, whereas the residues making contributions to the binding from the light chain were Tyr32L, Tyr50L, and Gly91L (Table S4, Figure 6A left).

Analysis of the per-residue contributions for the Bm10 antibody’s interaction with the M antigen revealed distinct sets of contributing residues for its two binding modes. In Binding Mode 1, the amino acids making significant contributions from the heavy chain were Trp52^H^, Arg78^H^, Gly119^H^, and His120^H^, and from the light chain were Leu70^L^, Tyr73^L^, Gln113^L^, Tyr115^L^, Tyr118^L^, and Arg120^L^ (Table S5, Figure 6B left). Whereas, for Binding Mode 2, a somewhat different set of residues contributed to the binding, including heavy chain residues Tyr51^H^, Trp52^H^, Trp66^H^, Ile69^H^, Arg78^H^, and His118^H^, and light chain residues Tyr49^L^, Tyr56^L^, Gln113^L^, Tyr115^L^, Tyr116^L^, Thr117^L^, Tyr118^L^, and Arg120^L^ (Table S6, Figure 6C left). Both the binding modes shared five residues that are involved in the binding of the M antigen (Trp52^H^, Arg78^H^, Gln113^L^, Tyr115^L^, and Arg120^L^), which implied that these residues formed an integral part of the Bm10 binding site, independent of the specific orientation of the antigen.

The nature of the interactions between the antigen and antibodies can be deduced by studying the energetic decomposition of MM-GBSA binding energies. The magnitude of the hydrophobic contributions is indicated by the sum of van der Waals and nonpolar desolvation energies, while that of the polar contribution is indicated by the sum of electrostatic and polar desolvation energies. The energetic decomposition revealed that the interactions in all three complexes were predominantly hydrophobic. For the YsT9.1-A interaction, the hydrophobic contribution was −19.6 ± 0.3 kcal/mol, which was significantly larger in magnitude than the electrostatic contribution of −1.7 ± 0.8 kcal/mol. Similarly, for the Bm10-M complex in Binding Mode 1, hydrophobic interactions contributed −20.1 ± 0.6 kcal/mol, while electrostatic contributions were a less favorable 2.3 ± 4.3 kcal/mol. In Binding Mode 2 of Bm10-M, the hydrophobic contribution was even more substantial at −28.7 kcal/mol, compared to an unfavorable electrostatic contribution of 2.1 kcal/mol. This strongly indicates that the burial of hydrophobic surfaces upon complex formation is a major driving force for stable binding in these systems.

The difference in contributions based on the antibody chains (heavy and light) were also analyzed and indicated a notable difference between the behavior of YsT9.1 and Bm10 antibodies. For the YsT9.1-A complex, the heavy chain (−16.7 ± 0.3 kcal/mol) provided a substantially larger contribution to the overall binding energy compared to the light chain (−4.6 ± 0.1 kcal/mol). Alternately, for the Bm10-M complex, the light chain contributed more favorably to binding (−11.7 ± 0.5 kcal/mol in Binding Mode 1 and −16.1 kcal/mol for Binding Mode 2) compared to the heavy chain (−6.1 ± 0.8 kcal/mol in Binding Mode 1 and −10.1 kcal/mol in Binding Mode 2). This implies distinct roles for the heavy and light chains in shaping the binding interfaces.

### 2.6. Contribution to Binding from the Perspective of the Antigen

To identify the monosaccharide residues within the oligosaccharide ligands that contribute most to the stability of the interaction (the epitope residues) the per-residue contributions of the antigens to the binding energy in the stable complexes were examined. The residues of the carbohydrate antigen are numbered 1 through 6, corresponding to the non-reducing end to the reducing end (Figure 6 right). The binding orientation of the antigen was notably different between the YsT9.1-A and Bm10-M complexes. In the Bm10 binding site (Figure 6B,C right), the antigen was bound in a flipped orientation relative to its position in YsT9.1 (Figure 6A right). Despite this difference in overall orientation, it was observed that across all three stable binding cases the residue at the non-reducing end of the antigen (Residue 1) consistently made the weakest contribution to the overall binding energy (Tables S7–S9). Rather, the significant interactions for stable binding primarily involved the internal and reducing-end residues of the hexasaccharide antigens.

### 2.7. Comparison of Preferred Binding Modes with Experimental Epitope Binding Data

Since the YsT9.1 and YsT9.2 mAbs were generated after immunization with killed cells of *Yersinia enterocolitica* O:9 that possesses an exclusively α1,2-linked Rha4NFo O-polysaccharide [13], it would be expected that their binding sites would not readily accept 1,3 linked elements characteristic of the M antigen. This is confirmed by the modeling data. Also, a 1,2-linked pentasaccharide was the most active oligosaccharide inhibitor of YsT9.1 binding to the A polysaccharide [15] and a hexasaccharide conjugated to protein was shown to be a better binder to YsT9.1 than either 1,2-linked penta- or tetrasaccharides [14]. This is fully consistent with the extended internal 1,2-linked internal sequences predicted by computational modeling reported here.

The internal trisaccharide Rha4NFo(1→3)Rha4NFo(1→2)Rha4NFo sequence of the M tetrasaccharide was a more effective inhibitor of Bm10 binding with the M-polysaccharide, while the Rha4NFo(1→2)Rha4NFo(1→3)Rha4NFo trisaccharide was weakly bound by it [17]. Consistent with calculations and predicted binding poses, the terminal non-reducing Rha4NFo of the M tetrasaccharide was not essential for binding Bm10, a fact supported when a mannose residue replaced the terminal non-reducing Rha4NFo residue [17]. Furthermore, a pentasaccharide (Rha4NFo(1→3)Rha4NFo(1→2)Rha4NFo(1→2)Rha4NFo(1→2)Rha4NFo) was a potent inhibitor of Bm10 [15]. This supports the prediction of calculations that a 1,3 linked disaccharide provides the primary focus of protein ligand binding and that additional Bm10- oligosaccharide interactions at the reducing end are possible.

## 3. Discussion

The study of antibody–antigen interactions is crucial for understanding the molecular basis of immune recognition and for developing targeted therapeutic strategies. In this work, we performed an extensive computational analysis of the binding of the four murine mAbs, YsT9.1, YsT9.2, Bm10, and Bm28, against the A and M epitopes of Brucella O-antigen. First, the 3D structures of the YsT9.2, Bm10, and Bm28 antibodies were generated by homology modeling. This was followed by docking analysis performed by using Vina-Carb, which generated a diverse set of binding poses for each antibody–antigen pair. Subsequently, the clustering of these poses provided distinct binding orientations for further investigation by MD simulations.

The MD simulations were performed in triplicates (100 ns each) and provided critical insights into the stability of the docked complexes through the Root Mean Square Fluctuation (RMSF) analysis. Significant variations were observed in the mobility of the antigens interacting with different antibodies and even between replicates of the same complex.

To further understand the basis of antibody–antigen preferences, the strength of their interactions was quantified through MM-GBSA binding energy calculations. There was a large variation in the binding strength for different poses ranging from −50.8 kcal/mol to 1.3 kcal/mol, reflecting the diversity of the binding interactions. A strong positive correlation was observed within a specific range of stable complexes (binding energy below −30 kcal/mol and RMSF below 2 Å), where lower RMSF (greater stability) corresponded to more favorable binding energies. However, beyond these values, the relationship became asymptotic, where poses with high mobility or RMSF values had low binding affinities, while stable or low RMSF poses (<1 Å) also had high binding affinities (<−30 kcal/mol). This correlation provided a key criterion for identifying stable binding poses from a large number of trajectories.

On the basis of this stringent stability criteria, only two complexes were deemed to be high affinity, the A antigen bound to YsT9.1 antibody (clusters ‘b’ and ‘d’) and the M antigen bound to Bm10 antibody (cluster ‘a’). This finding aligns well with experimental observations indicating YsT9.1’s preference for the A epitope and Bm10’s preference for the M epitope. However, no stable complexes were identified for YsT9.2 or for Bm28 antibodies with either A or M antigens. This could be a result of the lack of the antibody flexibility while docking, as rigid protein docking may not fully capture the conformational changes that can happen in the antibody upon antigen binding. Additionally, although the antibody homology models have high GMQE scores, they may not represent the true 3D structures of the antibodies. These shortcomings could potentially be addressed through flexible antibody docking or enhanced sampling techniques.

Detailed analysis of the stable YsT9.1-A and Bm10-M complexes through trajectory clustering revealed distinct, yet in the case of Bm10-M, multiple binding modes. For YsT9.1-A, the representative structures from the two stable clusters (‘b’ and ‘d’) occupied similar 3D space, suggesting a predominant binding mode for this interaction. On the other hand, the Bm10-M complex presented with two different binding modes from a singular stable cluster ‘a’. This observation suggests the potential for a single antibody to recognize an oligosaccharide epitope in more than one orientations.

Analysis of the per-residue contributions to binding energy shed light on the molecular determinants of specificity. In both the stable YsT9.1-A and Bm10-M complexes, a significant number of hydrophobic amino acids were found to contribute favorably to binding. The energetic decomposition confirmed the highly hydrophobic nature of these interactions, with van der Waals and nonpolar desolvation energies being the dominant driving forces, while electrostatic contributions were considerably smaller and even unfavorable in some cases for Bm10-M. The importance of hydrophobic contacts in antibody-carbohydrate antigen interactions have also been observed previously [25] and found in crystal structures of antibody–carbohydrate complexes [26,27,28].

The differences in the contribution to the binding energy from the heavy and the light chain of the antibodies were also observed. For the YsT9.1-A antigen complex, the heavy chain contributed more significantly to the binding energy with key contributions coming from residues Tyr32^H^, Tyr33^H^, Asp101^H^, and Tyr103^H^. On the contrary, in the case of Bm10-M antigen interactions the light chains were favored, but significant contributions came from both heavy and light chains. Interestingly, the two binding modes of Bm10-M complexes have five common residues (Trp52^H^, Arg78^H^, Gln113^L^, Tyr115^L^, and Arg120^L^) with significant contribution to binding. This suggests that there might be a conserved binding site present on the Bm10 antibody that can accommodate the M antigen in multiple orientations.

Analysis of the antigen’s contribution revealed an interesting pattern: in all identified stable complexes, the residue at the non-reducing end of the hexasaccharide had one of the lowest contributions to the overall binding energy. This implies that the antibodies have evolved to recognize an internal epitope rather than the terminal sequence of the polysaccharide antigen. Additionally, the antigens interacted with the antibodies in a completely different orientation, where the direction of binding was flipped between the YsT9.1-A and Bm10-M complexes. This suggests that while both antibodies recognize their preferred antigens, they do so by orienting the antigen differently within their binding pockets.

## 4. Methods

### 4.1. Brucella A and M Specific mAbs

#### 4.1.1. Monoclonal Antibodies

The four monoclonal antibodies studied here were generated in two separate experiments. YsT9.1 and YsT9.2 resulted from hybridomas generated from the spleens of mice immunized with the killed cells of *Yersinia enterocolitica* O:9 [13]. The cell wall of this bacterium contains an O-antigen of exclusively α1,2 linked D-Rha4NFo residues [29] and should therefore result in antibodies specific for α1,2 linked D-Rha4NFo sequences. Monoclonal antibodies Bm10 and Bm28 were generated from mice immunized with *Brucella melitensis* 16M [15], the prototypical *Brucella* variant expressing the M antigen. This variant expresses at least two repeating units of the M tetrasaccharide (Figure 1) [10].

#### 4.1.2. Sequencing of mAbs

The sequence of YsT9.1 Fab was determined by conventional protein sequencing methods [18]. The sequences of YsT9.2, Bm10, and Bm10 Fv domains were determined from mRNA extracted from hybridoma cells performed under contract (Fusion Antibodies, Belfast, UK). Protein sequences are provided in Supplementary Material (Figures S1–S6). Accession numbers for all sequence data deposited with GenBank and ABCD databases are listed at the end of the paper.

### 4.2. Modeling

#### 4.2.1. Structure Preparation

**Figure 7:**
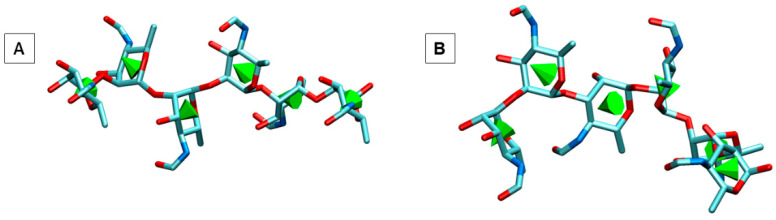
(**A**). The 3D structures of the A antigen: Rha4NFoα(1→2)Rha4NFoα(1→2)Rha4NFoα (1→2) Rha4NFoα(1→2)Rha4NFoα(1→2)Rha4NFo (top). (**B**). The 3D structures of the M antigen: Rha4NFoα(1→2)Rha4NFoα(1→3) Rha4NFoα(1→2)Rha4NFoα(1→2)Rha4NFoα(1→2) Rha4NFo (bottom), generated using the Carbohydrate Builder at GLYCAM-Web [31]. All the formamido groups in both the antigens are in Z conformation.

Murine mAbs Bm10, Bm28, YsT9.1, and YsT9.2 were considered for the analysis. The 3D structure of YsT9.1 as apo-protein is available in the PDB database with PDB ID 1MAM [18]. The 3D structures of the light and heavy chains of Bm10, Bm28, and YsT9.2 were generated using AlphaFold DB models through the SWISS-MODEL homology modeling server (swissmodel.expasy.org) [30]. All of the AlphaFold DB model structures had Global Model Quality Estimate (GMQE) quality scores greater than 0.83. GMQE is a quality estimate that integrates properties from target-template alignment and template structure to assess model quality. It aids in selecting optimal templates for modeling and is updated after model construction to enhance reliability. A homology structure with a GMQE score value above 0.7 is considered reliable. The Carbohydrate Builder at GLYCAM-Web (https://glycam.org/cb/ (accessed on 20 November 2023)) [31] was used to generate 3D structures of hexasaccharide fragments of the A and M epitopes of the Brucella O antigen (Figure 7). Chimera [32] was used to modify the hydroxyl group at O4 position to a formamido group in Z conformation.

#### 4.2.2. Docking and Clustering

All the input files for docking were prepared using AutoDock Tools [33] and docking was performed using Vina-Carb [34] with a grid box of dimensions x = 26, y = 26, and z = 38 Å, which was placed at the center of the complementarity determining regions (CDRs) of the antibody [35]. Default values were used, with the following exceptions: exhaustiveness = 80, num_modes = 100, chi_cutoff = 2, and chi_coeff = 1. All of the hydroxyl groups and glycosidic torsion angles were treated as flexible, while the protein was held rigid; the formamido group was also fixed in the Z (s-cis) conformation. Subsequently, the docked poses were clustered using the partitional k-means clustering algorithm provided by the Multi-scale modeling tools in the structural biology (MMTSB) [36] toolset. Only the highest-ranked pose from each cluster, as determined by Vina-Carb, was selected for subsequent MD simulation analysis.

#### 4.2.3. Molecular Dynamics (MD) Simulations

Antechamber was used to develop GAFF [37] partial atomic charges and force field parameters for the formamido group, ff14SB [38] parameters were employed for the amino acids, and GLYCAM06j [39] parameters for the sugar. All the complexes were solvated in a truncated octahedral box of TIP5P [40] water molecules with counter ions (Cl-) added to neutralize the charge, using the tLEAP module of AMBER. Previously [41], we have shown that the TIP5P model performs well with the GLYCAM/AMBER force field. All histidine residues were considered neutral with a hydrogen atom at the ε-position. The ionization states of the ionizable side chains (ASP, GLU, ARG, LYS) were set appropriately for a neutral pH, and kept in that state throughout the simulation. All simulations were performed using the Amber14 software suite [42]. The geometry was initially relaxed, followed by heating and equilibration following a recommended nine-step protocol [43]. This was followed by a 100 ns production run in triplicates for each complex. The atom coordinates were saved to the trajectory file every 10 ps or every 5000 simulation steps.

#### 4.2.4. Simulation Data Analysis

Root mean square deviation (RMSD) (Figure S7) and Root mean square fluctuation (RMSF) values were calculated for the antigen residues from the last 25 ns of the 100 ns of MD simulation, by extracting all of the last 2500 poses, using the cpptraj [44]. To identify different binding modes within the MD simulation, the trajectories were analyzed using MDAnalysis [45]. First, the independent MD simulations were merged and the interactions between antigen and antibody were quantified by computing distances between all atom pairs (interaction threshold <4.0 Å). For each frame, key features including mean and standard deviation of distances, interaction count, hydrogen bond count (distance < 3.5 Å and angle > 150°), and center of mass of the antigen were extracted. These features were normalized and subsequently clustered with K-Means (*n* = 3). Representative structures for each cluster were identified by finding the frame closest to the centroids of the clusters. Absolute binding affinity and per-residue contributions were estimated with the MMPBSA.py.MPI [46] module of AMBER employing the molecular mechanical (MM) interaction energies with a generalized Born approximation (MM-GBSA) for the desolvation free energies (igb = 2) [47] for 2500 snapshots extracted evenly from the last 25 ns of the 100 ns of MD simulation using the single trajectory method. The net binding energies were computed as the difference between those for the complex minus, those for the protein, and the ligand.

## 5. Conclusions

This study provides unique insights into the molecular basis of YsT9.1 and Bm10 antibody recognition of the *Brucella* O-antigen A and M epitopes. Stable binding modes were identified, interaction strengths were quantified, and crucial antibody and antigen residues involved in these interactions were objectively located. The observed dominance of hydrophobic forces and the differential contributions from the heavy and the light chains of the antibodies offers a deeper understanding of the recognition mechanism. The predicted binding modes and identified interaction hot spots may help to guide the design of synthetic glycomimetics, the engineering of epitope-specific antibodies, and possibly inform vaccine design.

## Data Availability

All the homology models and top docked poses from each cluster in this article can be freely accessed as PDB files at https://zenodo.org/records/15800977 (accessed on 29 June 2025) The DNA sequencing data are openly available at GenBank [https://www.ncbi.nlm.nih.gov/], accession numbers as follows: YsT9.2 VH Chain; PV749939-49944: VL Chain; PV749945-PV749951; Bm10 VH Chain; PV749910-PV749917: VL Chain; PV749918-PV749924; Bm28 VH Chain; PV749925-PV749932: VL Chain; PV749933- PV749938. Fv consensus amino acid sequences are available at the ABCD (AntiBodies Chemically Defined) database [https://web.expasy.org/abcd/ (accessed on 29 June 2025)], accession numbers as follows: YsT9.2 ABCD_BF026; Bm10 ABCD_BF027; Bm28 ABCD_BF028.

## Supplementary Materials

The following supporting information can be downloaded at: https://www.mdpi.com/article/10.3390/molecules30142906/s1, Figure S1: Graphical depiction of YsT9.2 Heavy Chain and CDR Loops; Figure S2: Graphical depiction of YsT9.2 Light Chain and CDR Loops; Figure S3: Graphical representation of the Bm10 VH chain and CDR Loops; Figure S4: Graphical representation of the Bm10 VL and CDR Loops; Figure S5: Graphical representation of the Bm10 VH and CDR Loops; Figure S6: Graphical representation of the VL Chain and CDR loops; Table S1: The number of clusters from each Antigen–Antibody docking simulation; Figure S7: The RMSD values, averaged over three independent MD simulations, of the top docked poses (a–f) from each cluster, obtained after docking the A antigen (left panels) or the M antigen (right panels) to murine Brucella antibodies YsT9.1 (A), YsT9.2 (B), Bm10 (C), and Bm28 (D); Table S2: RMSD between the ring atoms of representative structures of YsT9.1-A complex; Table S3: RMSD between the ring atoms of representative structures of Bm10-M complex; Table S4: Per-residue contribution of YsT9.1 antibody in complex with A antigen; Table S5: Per-residue contribution of Bm10 antibody in complex with M antigen in binding mode 1; Table S6: Per-residue contribution of Bm10 antibody in complex with M antigen in binding mode 2; Table S7: Per-residue contribution of the A antigen in complex with YsT9.1 antibody; Table S8: Per-residue contribution of the M antigen in complex with Bm10 antibody in binding mode 1; Table S9: Per-residue contribution of the M antigen in complex with Bm10 antibody in binding mode 2.

## Abbreviations

The following abbreviations are used in this manuscript:

PS	Polysaccharide
mAb	Monoclonal Antibody
Rha4NFo	4-formamido-4,6-dideoxy-⍺-D-mannopyrannose
GMQE	Global Model Quality Estimate
CDR	Complementarity-Determining Region
MMTSB	Multiscale Modeling Tools for Structural Biology
RMSF	Root Mean Square Fluctuation
RMSD	Root Mean Square Deviation
MD	Molecular Dynamics
MM	Molecular Mechanics
GBSA	Generalized Born and Surface Area
