# Supplementary material for: Towards Understanding the Basis of Brucella Antigen–Antibody Specificity
Source: Molecules. 2025 Jul 9;30(14):2906. doi: 10.3390/molecules30142906 (PMC12299769; doi:10.3390/molecules30142906)
Supplement: Supplementary file 1 [file molecules-30-02906-s001.zip › molecules-3704202-supplementary.pdf]

Supplementary

Material

mAb Sequencing

Sequencing was performed by Fusion Antibodies Ltd, Springbank Industrial Estate,Pembroke Loop Road, Belfast, N. Ireland. Total RNA was extracted from hybridoma cell pellets. cDNA was created from the RNA by reverse-transcription with an oligo(dT) primer. PCR reactions were set up using variable domain primers to amplify both the VH and VL regions of the three monoclonal antibodies (YsT9.2, Bm10 and Bm28) [1,2].

The VH and VL products were cloned into the Invitrogen sequencing vector pCR2.1 and transformed into TOP10 cells and screened by PCR for positive transformants. Selected colonies were picked and analyzed by DNA sequencing on an ABI3130xl Genetic Analyzer.

Sequence of Brucella A specific mAb YsT9.2 [1]:

V_H Consensus Amino Acid Sequence:
MEWSWVMLFILSGTAGVHS**QIQLQQSGAELARPGTSVKLSCKASGYTFTDYYINWVKQRTG QGLEWVGEISPGSGYTSYNEKFKGKATLTADKSSSTAYMQLSSLTSEDSAVYICARNSYYFD YWGQGTTLTVSS**ATTTAPSVYPLV

V_L Consensus Amino Acid Sequence:
MKLPVRLLVLWIRETNG**DVVMTQTPLTLSVTIGQPASISCKSSQSLLDSDGKTYLHWLLQRPG QSPKRLIYLVSTLDSGVPDRFTGSGSGTDFTLKISRVEAEDLGVYYCWQGTHFPLTFGAGTKL ELK**RADAAPTVSIFPPSSEQLTSGGASVVCFLNNFYPK

The variable domains are highlighted in bold. The Complementarity Determining Regions (CDRs) are highlighted in yellow as determined by the IMGT numbering system.

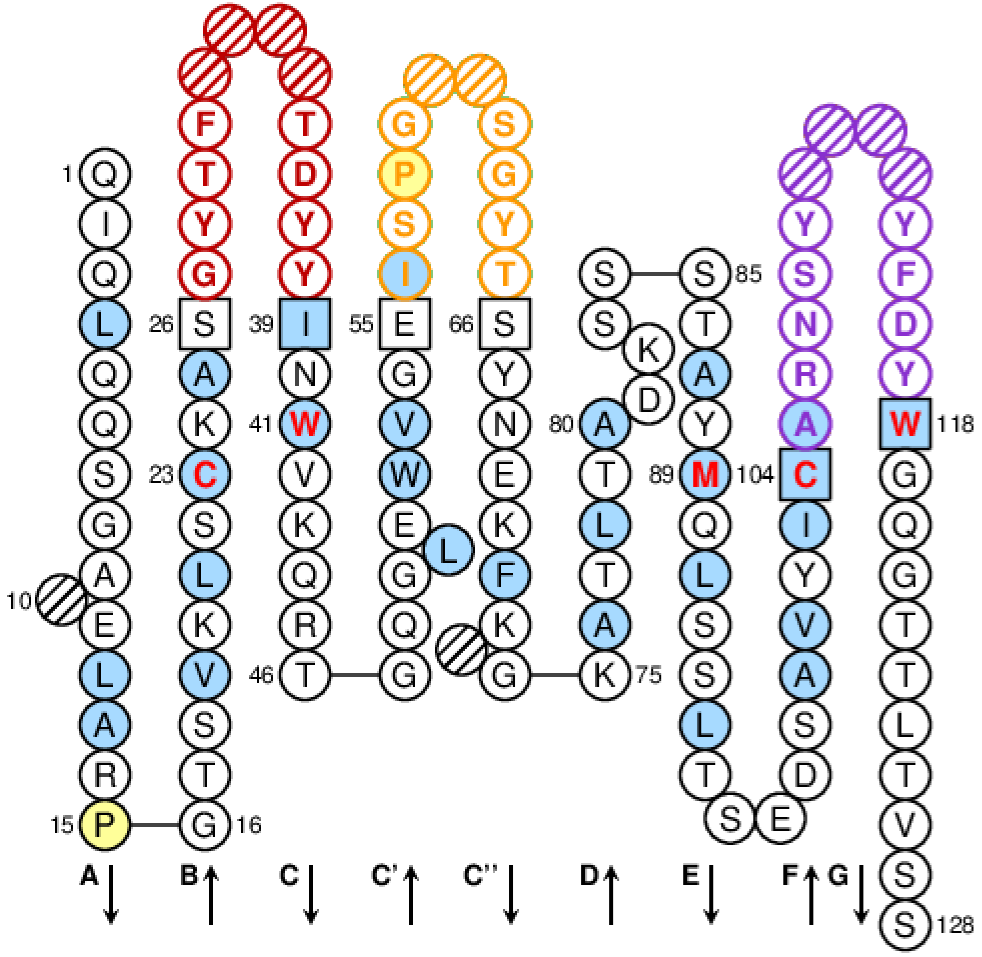

Figure S1. Graphical depiction of YsT9.2 Heavy Chain and CDR Loops

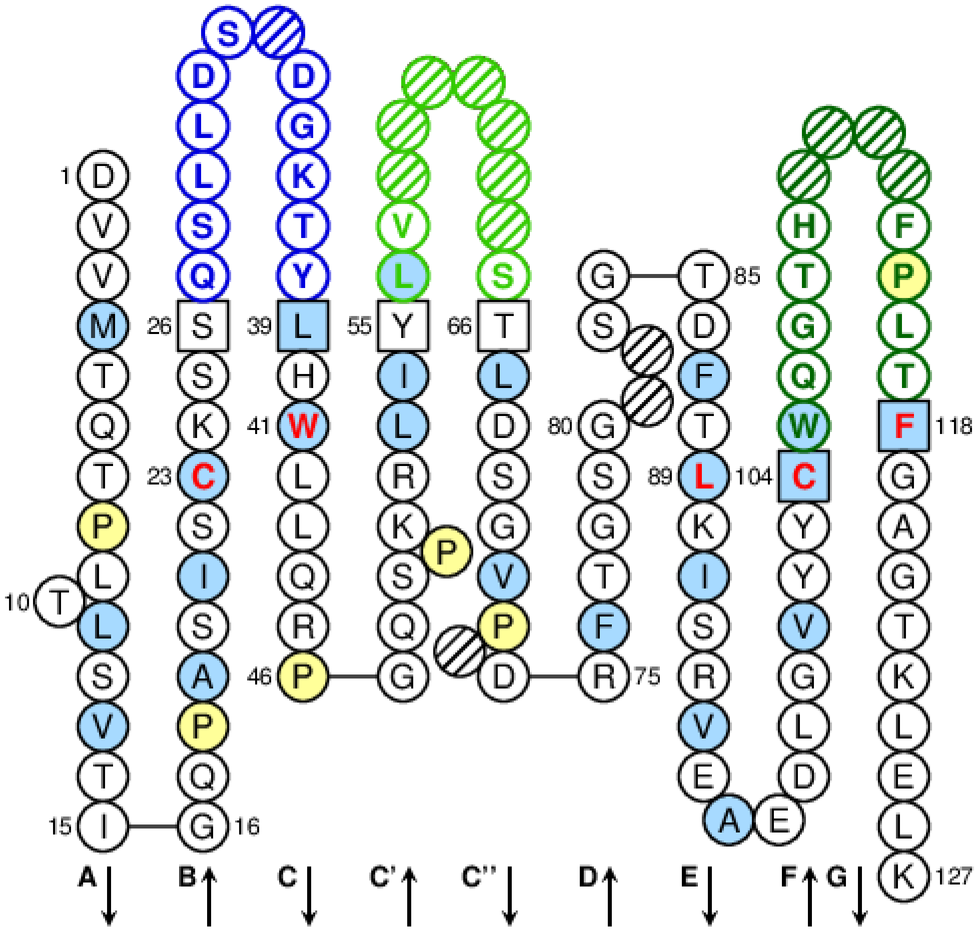

Figure S2. Graphical depiction of YsT9.2 Light Chain and CDR Loops

Sequence of Brucella M specific mAb Bm10 [2]:

V_H Consensus Amino Acid Sequence:
MGWSCIMLFLVATATGVHS**QVQLQQPGAELVRPGASVKLSCKASGYSFTSYWMNWVKQRPGQGLEWIGIIHPSDSETRLNQKFKDKATLTVDKSSSTAYMQLSSPTSEDSAVYYCSRHGHMDYWGQGTSVTVSS**ATTTAPPVYPLA

V_L Consensus Amino Acid Sequence:
MVLLLLLLWVSGTCG**DIVMSQSPSSLAVSVGEKVTMSCKSSQSLLYSNDQKNYLAWYQQKPGQSPKLLIYWASTRESGVPDRFTGSGSGTDFTLTISSVKAEDLAVYYCQQYYTYPRTFGGGTKLEIK**RADAAPTVSIFPPSSEQLTSGGASVVCFLNNFYPK

The variable domains are highlighted in bold; the Complementarity Determining Regions (CDRs) are highlighted in yellow.

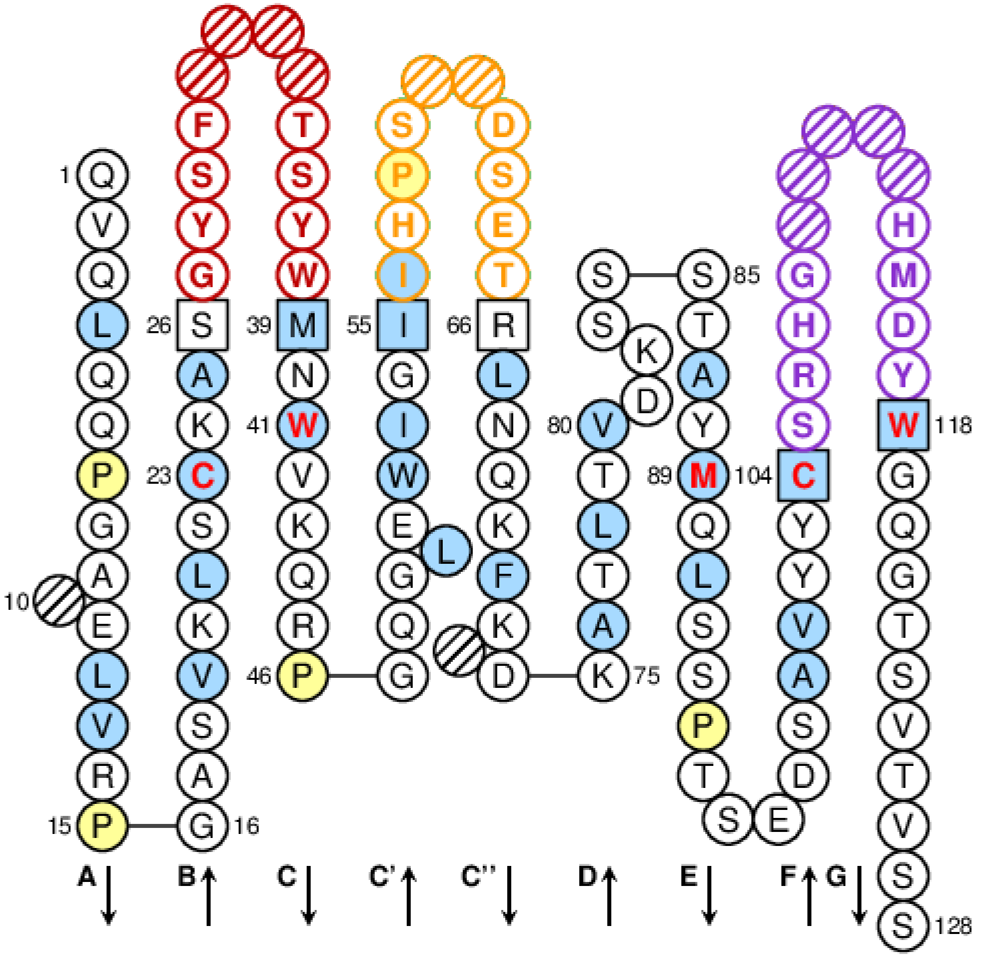

Figure S3. Graphical representation of the Bm10 VH chain and CDR Loops

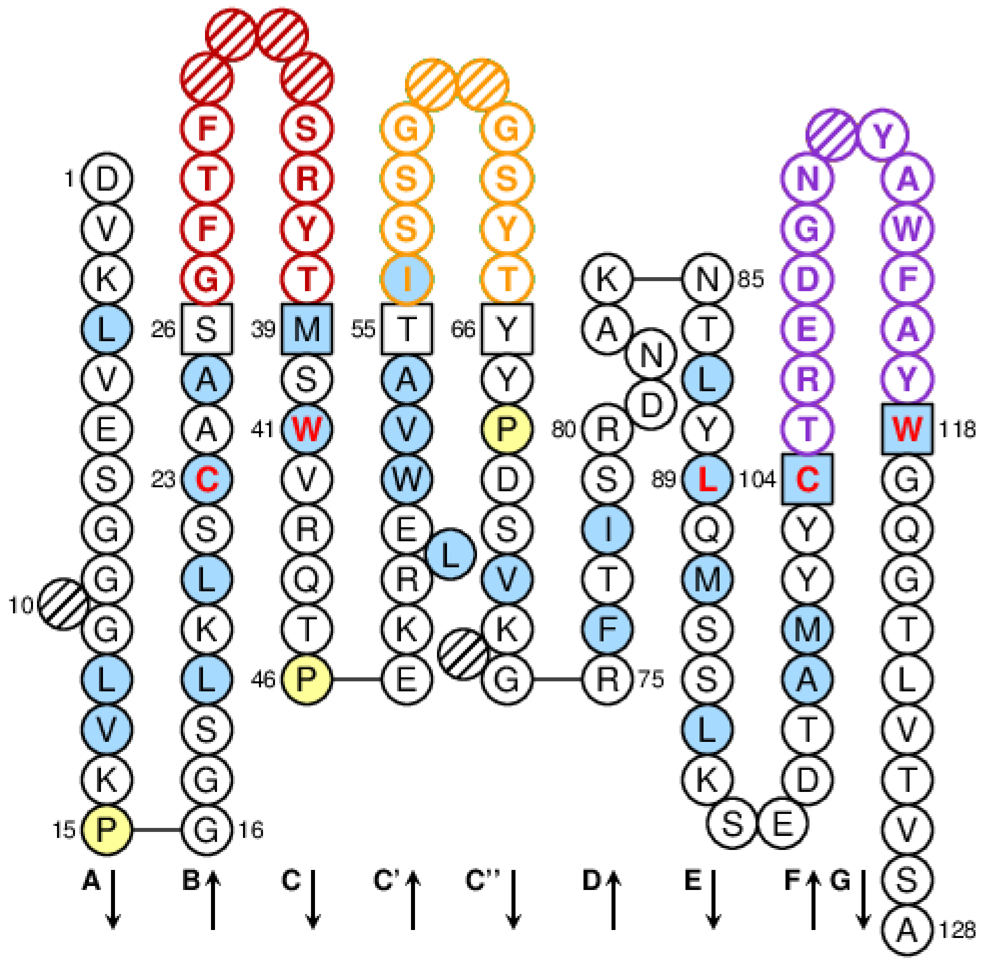

Figure S4. Graphical representation of the Bm10 VL and CDR Loops

Sequence of Brucella M specific mAb Bm28 [2]:

V_H Consensus Amino Acid Sequence:
MDFGLSWVFLVLTLKGVQC**DVKLVESGGGLVKPGGSLKLSCAASGFTFSRYTMSWVRQTPE**
KRLEWVATISSGGSYTYYPDSVKGRFTISRDNAKNTLYLQMSSLKSEDTAMYYCTREDGNYA
WFAYWGQGTLVTVSAATTTAPSVYPLA

V_L Consensus Amino Acid Sequence:
MKLPVRLLVLWIRETNG**DVVMTQTPLTLSVTIGQPASISCKSSQSLLDSEGKTYLHWFLQRPG**
QSPKRLIYLVSKLDSGVPDRFTGSGSGTDFTLKISRVEAEDLGVYYCWQGTHFPYTFGGGTK
LEIRRADAAPTVSIFPPSSEQLTSGGASVVCFLNNFYPR

The variable domains are highlighted in bold; the Complementarity Determining Regions (CDRs) are highlighted in yellow.

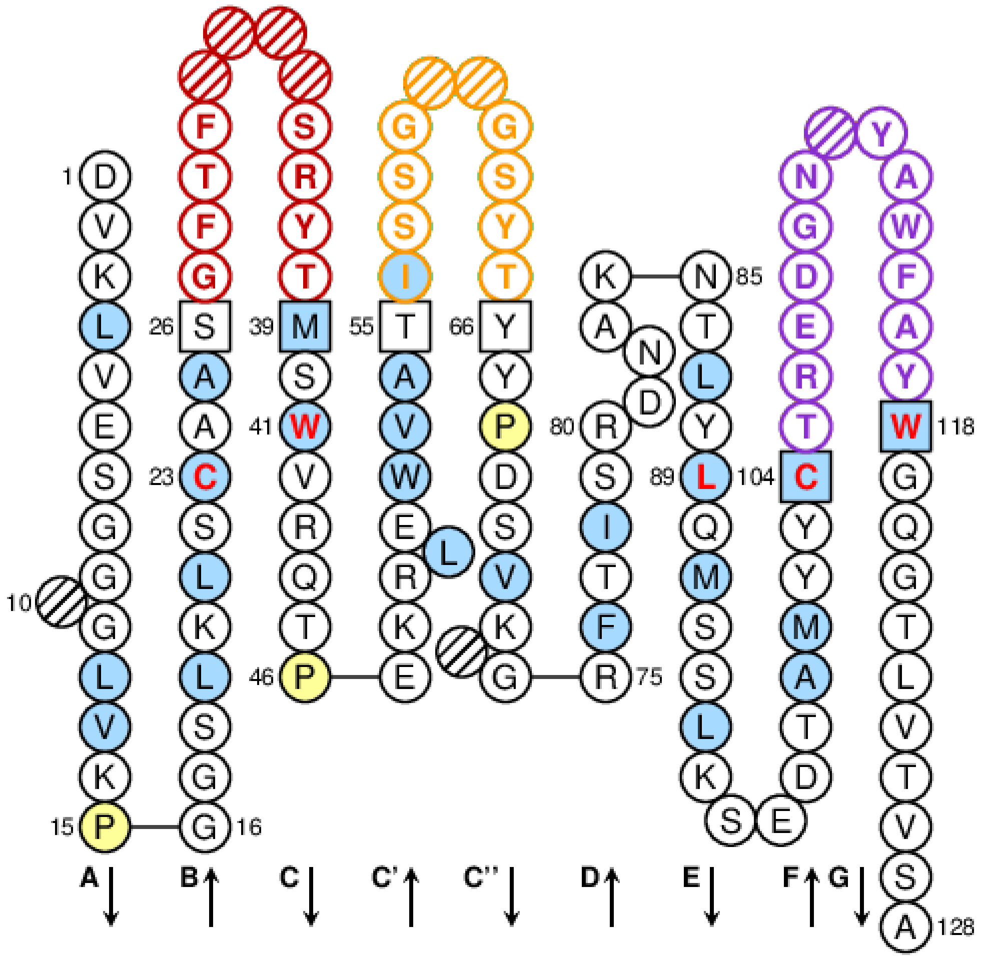

Figure S5. Graphical representation of the Bm10 VH and CDR Loops.

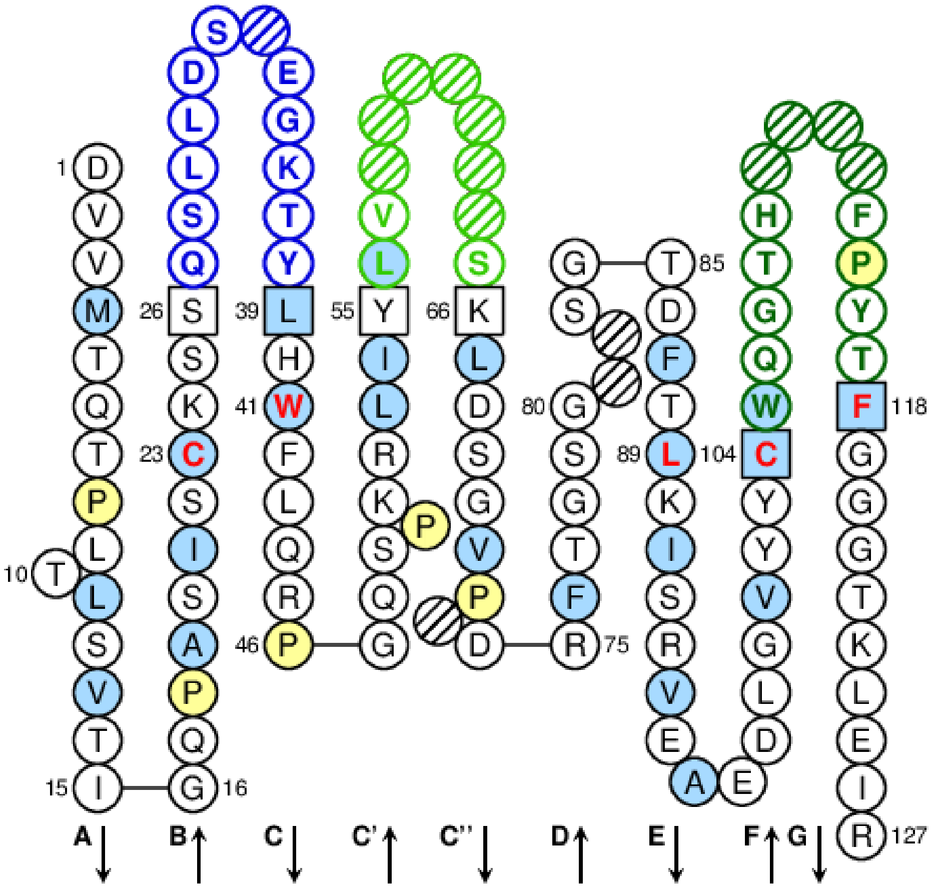

Figure S6. Graphical representation of the VL Chain and CDR loops.

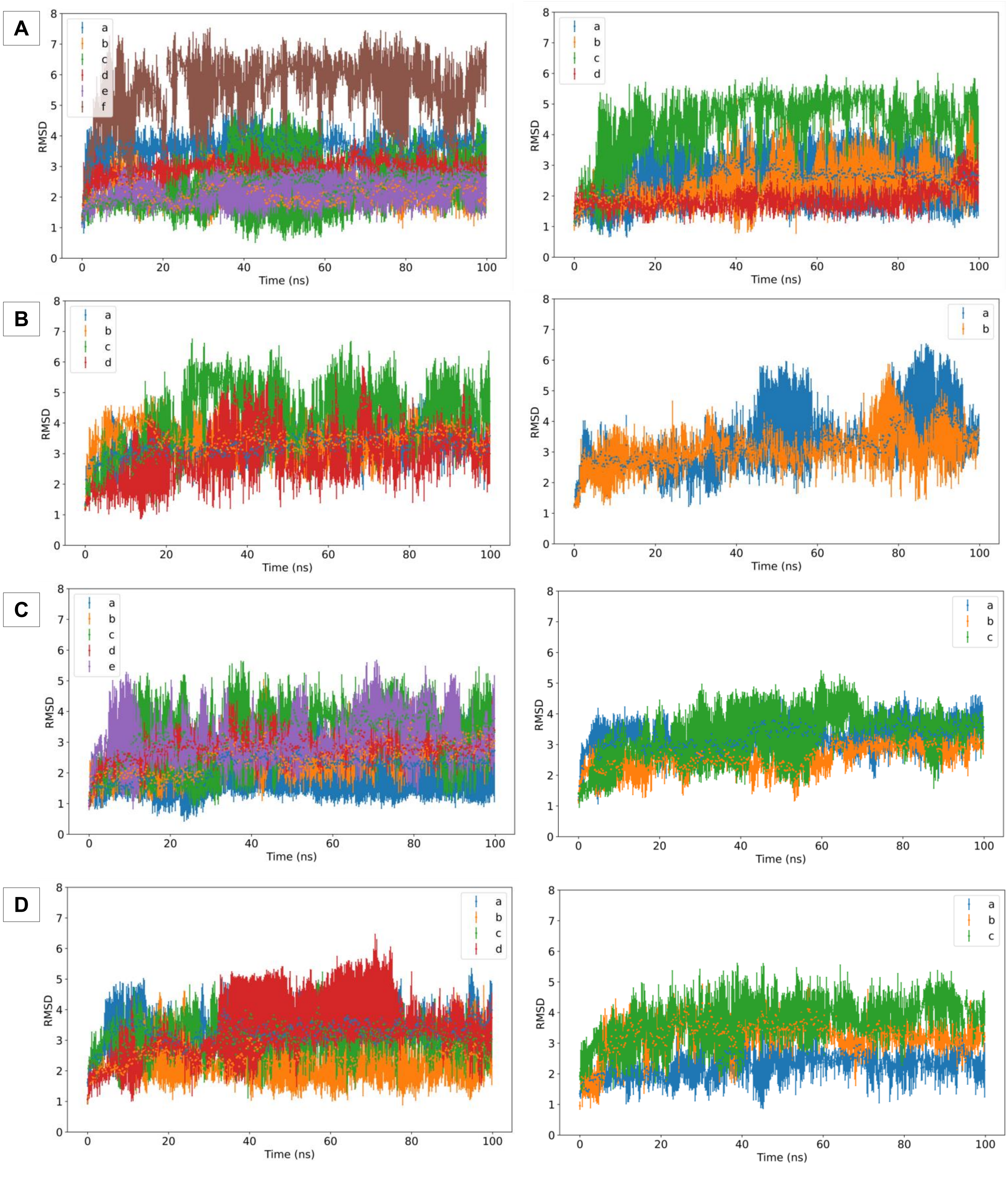

A
B
C
D
RMSD
Time (ns)
a
b
c
d
e
f

Figure S7. The RMSD values, averaged over three independent MD simulations, of the top docked poses (a-f) from each cluster, obtained after docking the A antigen (left panels) or the M antigen (right panels) to murine *Brucella* antibodies YsT9.1 (A), YsT9.2 (B), Bm10 (C) and Bm28 (D).

Table S1. The number of clusters from each Antigen-Antibody docking simulation.

Antibody	Antigen	No. of clusters
YsT9.1	A	6
	M	4
YsT9.2	A	4
	M	2
Bm10	A	5
	M	3
Bm28	A	4
	M	3

Table S2. RMSD values[a] between the ring atoms of representative structures of YsT9.1-A complex.

Structure	1	2	3
1	0	0.8	2.1
2	2.1	0	2.1
3	2.1	0.8	0

[a]Å.

Table S3. RMSD values[a] between the ring atoms of representative structures of Bm10-M complex.

Structure	1	2	3
1	0	1.4	3.9
2	3.6	0	3.6
3	3.9	1.4	0

[a]Å.

Table S4. Per-residue interaction energy contributions[a,b] of YsT9.1 antibody in complex with A antigen.

Residue Name	Residue Number	CDR	vdW	Electrostatic	Polar Desolvation	Nonpolar Desolvation	Total
TYR	32	H	-1.4 (0.2)	-1.7 (0.1)	1.6 (0.1)	-0.2 (0.0)	-1.7 (0.1)
TYR	33	H	-1.7 (0.1)	-2.1 (0.0)	1.5 (0.0)	-0.2 (0.0)	-2.4 (0.1)
ASP	101	H	0.0 (0.1)	-15.0 (0.4)	10.8 (0.5)	-0.1 (0.0)	-4.3 (0.2)
TYR	103	H	-5.0 (0.1)	-0.6 (0.1)	2.2 (0.1)	-0.6 (0.0)	-4.1 (0.1)
PRO	105	H	-3.3 (0.1)	0.2 (0.1)	0.3 (0.1)	-0.5 (0.0)	-3.2 (0.0)
ALA	106	H	-1.1 (0.1)	0.9 (0.0)	-0.7 (0.0)	-0.1 (0.0)	-1.0 (0.1)
TYR	32	L	-3.1 (0.2)	0.0 (0.1)	1.6 (0.0)	-0.5 (0.0)	-2.1 (0.1)
TYR	50	L	-1.6 (0.1)	-0.2 (0.1)	0.5 (0.1)	-0.2 (0.0)	-1.4 (0.1)
GLY	91	L	0.0 (0.0)	-4.3 (0.2)	3.2 (0.2)	-0.1 (0.0)	-1.1 (0.0)

[a]All binding energy values in kcal/mol. [b]Values in parenthesis are the standard deviations over six MD simulations.

Table S5. Per-residue interaction energy contributions[a,b] for Bm10 antibody in complex with M antigen in binding mode 1.

Residue Name	Residue Number	CDR	vdW	Electrostatic	Polar Desolvation	Nonpolar Desolvation	Total
TRP	52	H	-2.5 (0.1)	0.1 (0.1)	0.3 (0.1)	-0.4 (0.0)	-2.5 (0.1)
ARG	78	H	-1.5 (0.4)	-9.2 (3.1)	9.8 (2.9)	-0.3 (0.1)	-1.2 (0.7)
GLY	119	H	-1.6 (0.2)	0.0 (0.1)	0.6 (0.1)	-0.2 (0.0)	-1.2 (0.3)
HIE	120	H	-1.3 (0.2)	-1.0 (0.1)	1.2 (0.4)	-0.2 (0.1)	-1.3 (0.0)
LEU	70	L	-1.1 (0.0)	0.1 (0.0)	0.0 (0.1)	-0.1 (0.0)	-1.1 (0.1)
TYR	73	L	-2.8 (0.3)	-0.2 (0.0)	1.1 (0.2)	-0.3 (0.1)	-2.2 (0.3)
GLN	113	L	-0.5 (0.1)	-1.5 (0.3)	0.2 (0.2)	-0.0 (0.0)	-1.8 (0.2)
TYR	115	L	-3.5 (0.1)	-0.5 (0.2)	1.7 (0.1)	-0.4 (0.0)	-2.6 (0.0)
TYR	118	L	-1.5 (0.0)	-4.0 (0.3)	4.8 (0.0)	-0.3 (0.0)	-1.1 (0.2)
ARG	120	L	-1.3 (0.0)	-15.2 (0.1)	13.9 (0.2)	-0.2 (0.0)	-2.9 (0.2)

[a]kcal/mol. [b]Values in parenthesis are the standard deviations over two MD simulations.

Table S6. Per-residue interaction energy contributions[a,b] of Bm10 antibody in complex with M antigen in binding mode 2.

Residue Name	Residue Number	CDR	vdW	Electrostatic	Polar Desolvation	Nonpolar Desolvation	Total
TYR	51	H	-1.1	-1.3	0.9	-0.2	-1.7
TRP	52	H	-1.9	-1.3	0.8	-0.3	-2.7
TRP	66	H	-0.8	0.2	-0.3	-0.1	-1.0
ILE	69	H	-1.3	0.1	-0.1	-0.2	-1.5
ARG	78	H	-1.6	-5.8	6.7	-0.3	-1.0
HIE	118	H	-4.0	-1.6	3.2	-0.4	-2.8
TYR	49	L	-1.5	-0.8	1.6	-0.3	-1.0
TYR	56	L	-1.5	-0.1	0.5	-0.2	-1.3
GLN	113	L	-0.4	-2.0	1.0	0.0	-1.5
TYR	115	L	-3.4	-3.1	3.1	-0.5	-3.9
TYR	116	L	-1.8	-4.3	4.3	-0.2	-2.0
THR	117	L	-2.0	-1.1	1.1	-0.2	-2.2
TYR	118	L	-2.5	-0.7	1.8	-0.3	-1.7
ARG	120	L	-1.5	-5.2	4.5	-0.2	-2.5

[a]kcal/mol. [b]Values are derived from one MD simulation run.

Table S7. Per-residue interaction energy contributions[a,b] of the A antigen in complex with YsT9.1 antibody.

Oligosaccharide Residue Number	vdW	Electrostatic	Polar Desolvation	Nonpolar Desolvation	Total
6	-6.6 (0.4)	-9.9 (0.6)	16.1 (1.1)	-1.2 (0.1)	-1.7 (0.2)
5	-2.8 (0.2)	-2.1 (0.5)	3.8 (0.5)	-0.3 (0.0)	-1.4 (0.2)
4	-7.0 (0.2)	-3.8 (0.6)	8.3 (0.7)	-1.1 (0.0)	-3.6 (0.2)
3	-7.3 (0.1)	-16.8 (0.3)	18.8 (0.3)	-1.3 (0.0)	-6.5 (0.1)
2	-1.3 (0.1)	-1.6 (0.3)	3.1 (0.3)	-0.1 (0.0)	0.0 (0.1)
1	-2.2 (0.3)	0.5 (0.4)	1.8 (0.4)	-0.5 (0.1)	-0.3 (0.2)

[a]kcal/mol. [b]Values in parenthesis are the standard deviations over six MD simulations.

Table S8. Per-residue interaction energy contributions[a,b] of the M antigen in complex with Bm10 antibody in binding mode 1.

Oligosaccharide Residue Number	vdW	Electrostatic	Polar Desolvation	Nonpolar Desolvation	Total
6	-5.4 (0.6)	-17.5 (2.2)	18.9 (1.3)	-1.1 (0.0)	-5.1 (0.6)
5	-9.4 (0.2)	-15.5 (0.4)	17.0 (0.3)	-1.5 (0.0)	-9.4 (0.3)
4	-3.5 (0.0)	1.1 (0.6)	2.3 (0.5)	-0.5 (0.0)	-0.6 (0.1)
3	-6.5 (0.1)	-14.4 (0.5)	14.7 (0.3)	-1.2 (0.0)	-7.3 (0.2)
2	-2.9 (0.1)	0.1 (1.3)	1.9 (1.3)	-0.7 (0.1)	-1.6 (0.1)
1	-3.0 (0.8)	5.8 (1.8)	-3.5 (1.8)	-0.6 (0.2)	-1.4 (1.4)

[a]All binding energy values in kcal/mol. [b]Values in parenthesis are the standard deviations over two MD simulations.

Table S9. Per-residue interaction energy contributions[a,b] of the M antigen in complex with Bm10 antibody in binding mode 2.

Residue Number[3]	vdW	Electrostatic	Polar Desolvation	Nonpolar Desolvation	Total
6	-6.0	-11.6	15.7	-1.0	-2.8
5	-9.1	-9.9	14.3	-1.5	-6.2
4	-4.5	0.2	2.2	-0.6	-2.7
3	-7.8	-6.5	12.3	-1.4	-3.4
2	-5.2	-4.4	5.9	-1.0	-4.6
1	-1.5	3.2	-1.4	-0.3	0.1

[a]All binding energy values in kcal/mol. [b]Values are derived from one MD simulation run.

References

1. Bundle, D.R.; Gidney, M.A.J.; Perry, M.B.; Duncan, J.R.; Cherwonogrodzky, J.W. Serological confirmation of *Brucella abortus* and *Yersinia enterocolitica* O:9 O-antigens by monoclonal antibodies. *Infect. Immun*., **1984**, *46*, 389-393.
2. Bundle, D. R.; Cherwonogrodzky, J. W.; Gidney, M. A. J.; Meikle, P. J.; Perry, M. B.; Peters, T. Definition of *Brucella* A and M epitopes by monoclonal typing reagents and synthetic oligosaccharides. *Infect. Immun*., **1989,** *57,* 2829–2836.
